# Impact of neck percutaneous interferential current sensory stimulation on swallowing function in patients with Parkinson's disease: A single-arm, open-label study protocol

**DOI:** 10.1016/j.conctc.2023.101158

**Published:** 2023-06-10

**Authors:** Masahiro Nakamori, Megumi Toko, Hidetada Yamada, Yuki Hayashi, Kohei Yoshikawa, Mineka Yoshikawa, Toshikazu Nagasaki, Aya Hiraoka, Yoshitaka Shimizu, Yukio Mikami, Hirofumi Maruyama

**Affiliations:** a Department of Clinical Neuroscience and Therapeutics, Hiroshima University Graduate School of Biomedical and Health Sciences, Hiroshima, Japan; b Department of Rehabilitation Medicine, Hiroshima University Hospital, Hiroshima, Japan; c Department of Advanced Prosthodontics, Hiroshima University Graduate School of Biomedical and Health Sciences, Hiroshima, Japan; d Department of Oral and Maxillofacial Radiology, Hiroshima University Graduate School of Biomedical and Health Sciences, Hiroshima, Japan; e Department of Dental Anesthesiology, Hiroshima University Graduate School of Biomedical and Health Sciences, Hiroshima, Japan

**Keywords:** Parkinson's disease, Deglutition disorders, Cough, Hypesthesia, Aspiration, Interferential current sensory stimulation

## Abstract

**Background:**

Parkinson's disease (PD) can lead to swallowing dysfunction, resulting in aspiration pneumonia. Among the types of swallowing disorders, a characteristic and serious problem associated with PD is silent aspiration due to pharyngeal and laryngeal hypoesthesia.

**Methods:**

This single-arm, open-label study aims to evaluate the effectiveness of percutaneous neck interferential current sensory stimulation in enhancing swallowing function in patients with PD. The efficacy and safety of percutaneous neck interferential current sensory stimulation will be investigated for patients diagnosed with PD, based on the Movement Disorder Society criteria, of Hoehn–Yahr stages 2–4. The patients will receive neck percutaneous interferential current sensory stimulation for 20 min twice a week for 8 weeks using a Gentle Stim® (FoodCare Co., Ltd., Kanagawa, Japan) device. Once the intervention is initiated, evaluations will be performed every 4 weeks for a 16-week period. The primary endpoint to be assessed is the proportion of patients with normal cough with 1% citric acid at the end of the intervention (8 weeks after intervention initiation) compared with that at the beginning. This clinical trial will examine the usefulness of percutaneous neck interferential current sensory stimulation in patients with PD. In addition, this study will use novel instruments, such as multichannel surface electromyography and electronic stethoscope, to evaluate swallowing function.

**Discussion:**

This novel evaluation can provide insights into dysphagia in patients with PD and the usefulness of percutaneous neck interferential current stimulation. This exploratory study is limited by its single-arm, open-label design and small size.

**Trial registration number:**

jRCTs062220013; pre-results.

## Highlights

• Presents a protocol for a single-arm, open-label exploratory study.
• Aims to evaluate swallowing function in patients with PD.
• Aims to identify effects of percutaneous interferential current sensory stimulation.
• The study will use multichannel surface electromyography and electronic stethoscope.

## 1 Introduction

Neurological diseases can cause swallowing dysfunction that can lead to aspiration pneumonia, resulting in increased patient mortality and morbidity and healthcare costs. Parkinson's disease (PD) is a major neurodegenerative disease with a rapidly rising incidence among aging societies globally, a phenomenon that is called the “Parkinson pandemic” [1]. Aspiration pneumonia is the most common cause of death in patients with PD. Therefore, it is essential to better evaluate and rehabilitate patients with aspiration pneumonia. PD leads to the following types of swallowing disorders: abnormal transport from the oral cavity and pharynx [2], delayed swallowing reflex [3], pharynx residue [4], and others. Among these, hypoesthesia of the pharynx and larynx is particularly serious and is characteristic of PD [4]. The cough test is a simple and useful method for assessing silent aspiration risk [5]. Strengthening swallowing-associated muscles, such as the tongue, soft palate, and suprahyoid muscles, is a useful and established method for swallowing rehabilitation. However, no effective method for preventing silent aspiration and improving sensory function has been established yet.

Recently, several instruments have been developed to stimulate and modulate neurological functions. Percutaneous electrical stimulation of the neck has been found to improve neuromuscular function. To evoke muscle contractions, several types of stimulation methods involving pulsed current stimulation have been found to be effective against various types of dysphagia [6,7] and are widely applied in clinical practice [8]. A drawback of the method is that muscle contraction is painful and invasive. Interferential current sensory stimulation may also be used for treating dysphagia because activation of peripheral sensory nerves in the pharynx and larynx can enhance sensitivity and protect the airway from aspiration [9]. Reports have shown that electrical stimulation devices may improve swallowing function without muscle contraction [10,11]. In addition to motor nerve stimulation for promoting muscle contraction, the stimulation of sensory nerves may be key for improving dysphagia.

Interferential currents can extend to deeper tissues and are more comfortable for the patient than pulsed currents [12]. Thus, they are expected to stimulate sensory nerves in deep layers of the pharynx and larynx without causing discomfort. For these reasons, interferential current stimulation has the potential for reducing dysphagia [13]. Interferential current stimulation reportedly enhances saliva production [14], shortens pharyngeal latency, increases swallowing frequency in healthy men, and increases airway sensitivity [13]. Moreover, for patients with dysphagia, neck percutaneous interferential current sensory stimulation may improve airway defense and nutrition [15].

However, the mechanism underlying swallowing dysfunction improvement in patients with neurological diseases remains unknown, as are the specific effects of device use. Therefore, we aimed to identify the effects of percutaneous interferential current sensory stimulation of the neck on swallowing function of patients with PD via comprehensive swallowing evaluation through videofluoroscopic examination. Sensory functions of the pharynx and larynx will be assessed via a cough test. Moreover, novel instruments, such as multichannel surface electromyography and electronic stethoscope, will be used to evaluate swallowing function in patients with PD.

## 2 Material and methods

### 2.1 Ethics approval

This study was approved by the Hiroshima University Certified Review Board (approval number: CRB6180006). It has been registered with jRCT (jRCTs062220013). Written informed consent will be obtained from all patients or their relatives.

### 2.2 Study design

The first draft of the study protocol, of a single-arm, open-label study, was finalized on February 28, 2022, and the draft adhered to the SPIRIT reporting guidelines [16]. The second amended draft was finalized on December 31, 2022. We designed the study to determine the efficacy and safety of percutaneous neck interferential current sensory stimulation for patients with PD, diagnosed according to the Movement Disorder Society criteria, of Hoehn–Yahr stages 2–4 at Hiroshima University Hospital [17]. This study aims to identify the factors associated with swallowing dysfunction and determine the safety of neck percutaneous interferential current sensory stimulation. Therefore, neither randomization nor blinding was used in this study design. The patient inclusion and exclusion criteria are presented in Table 1.

**Table 1:** Eligibility criteria.

Inclusion criteria
1. Diagnosis of clinically probable or established PD based on the Movement Disorder Society criteria 2. PD of Hoehn–Yahr stages 2–4 at the time of registration 3. Levodopa dosage remaining unchanged for more than 1 month 4. Age >19 and < 86 years 5. Able to visit the hospital twice a week 6. Able to provide written informed consent

**Exclusion criteria**
1. Pacemaker or implantable defibrillator 2. Ongoing treatment with deep brain stimulation 3. Currently pregnant or trying to become pregnant 4. Currently diagnosed with or having a history of head or neck cancer 5. Active pneumonia 6. History of swallowing rehabilitation 7. Exclusion considered appropriate by doctors

**Fig. 1:**
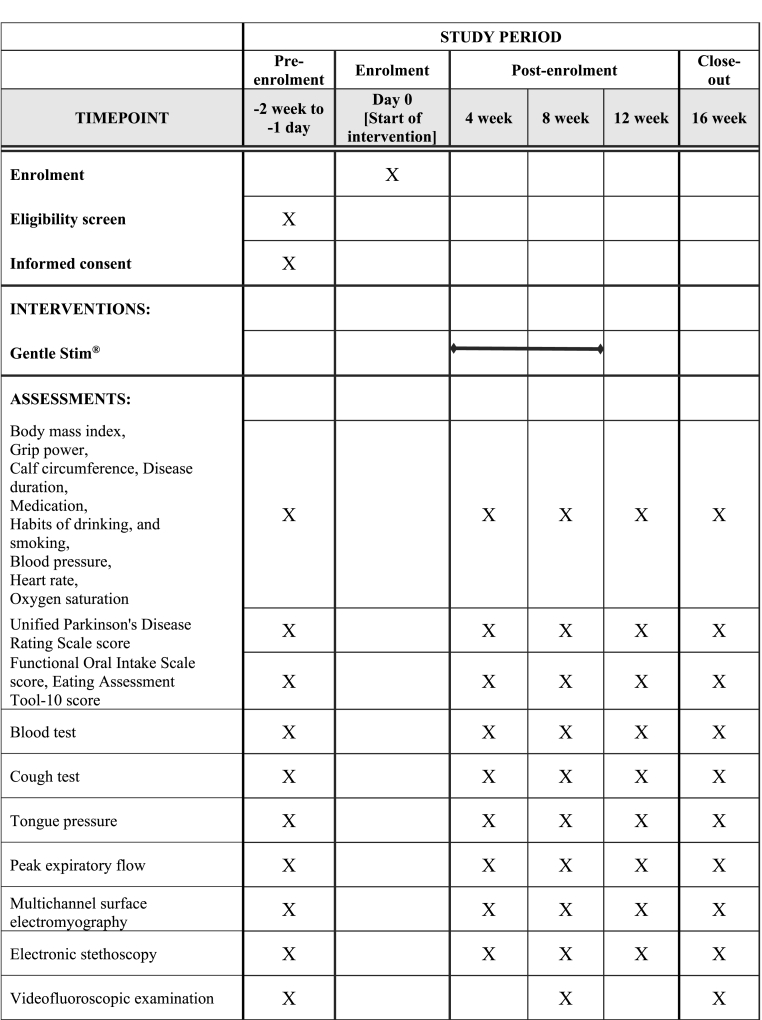
SPIRITschedule of enrollment, interventions, and assessments.

**Fig. 2:**
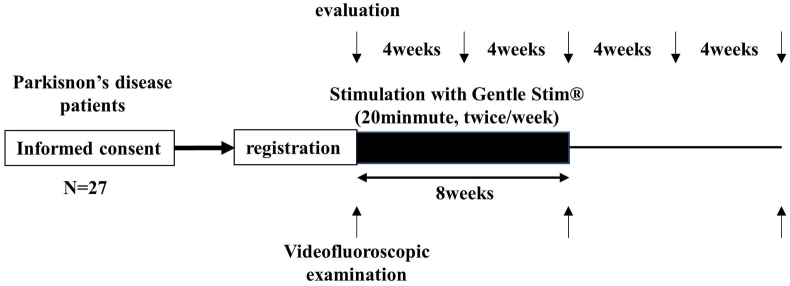
Schema of study timeline.

Fig. 1 shows the SPIRIT schedule of enrollment, interventions, and assessments. Schematics outlining the study timeline are presented in Fig. 2. The patients will undergo neck interferential current sensory stimulation for 20 min twice a week for 8 weeks. A Gentle Stim® (FoodCare Co., Ltd., Kanagawa, Japan) device will be used for stimulation. Pads will be attached to the front of the neck to stimulate the swallowing-related (superior laryngeal) nerve. The swallowing reflex interferential current (50 Hz) stimulation threshold is lower than the pulse stimulation; therefore, patients may scarcely feel the stimulation. The patients will be stimulated according to a unified protocol involving maximum stimulation current below the threshold at which the patient perceives electrical stimulation, ranging from 2.0 mA to 2.5 mA. Stimulation will be performed regularly and repetitively.

Evaluations will be performed every 4 weeks from the beginning of the intervention to 16 weeks post-intervention initiation. Videofluoroscopic examinations will be performed every 8 weeks from the beginning of the intervention to 16 weeks post-intervention initiation. The primary, secondary, and exploratory endpoints are listed in Table 2.

**Table 2:** Primary, secondary, and exploratory endpoints.

Primary endpoint
The proportion of patients with normal cough reflex after the 1% citric acid cough challenge at the end of the intervention (8 weeks from the initiation). In this study, a normal cough reflex is defined as coughing five or more times 1 min after the consumption of 1% citric acid.

**Secondary endpoints**
1. Proportion of patients with a normal cough reflex after challenge with 1% citric acid 8 weeks after the last intervention (16 weeks from the initiation of intervention) 2. Proportion of patients with a cough reflex after challenge with 1% citric acid within the first 30 s after the last intervention (8 weeks from the initiation of intervention) 3. Proportion of patients with a cough reflex after 1% citric acid challenge within the first 30 s at 8 weeks after the end of the last intervention (16 weeks from the initiation of intervention) 4. Proportion of patients with normal swallowing status 8 weeks after the end of the intervention (16 weeks after intervention initiation) (normal swallowing status is defined as a Functional Oral Intake Scale score of 7 and an Eating Assessment Tool score of <3) 5. Proportion of patients with a normal swallowing status 8 weeks after the end of the intervention (16 weeks after intervention initiation) 6. Proportion of aspiration and penetration via videofluoroscopic examination at the end point of the intervention (8 weeks after intervention initiation) 7. Proportion of those with aspiration and penetration revealed via videofluoroscopic examination 8 weeks after the end of the intervention (16 weeks after the initiation of the intervention) 8. Incidence of pneumonia onset during the 16-week period after the initiation of the intervention

**Exploratory endpoints**
1. Changes in bolus passing determined by temporal videofluoroscopic examination 2. Changes in multichannel surface electromyography findings 3. Changes in tongue pressure 4. Changes in peak expiratory flow 5. Changes in swallowing sound

**Safety**
1. Skin symptoms at the region of electrode attachment 2. Exacerbation of neurological symptoms due to electrical stimulation of the head and neck

### 2.3 Sample size

We calculated the required sample size according to preliminary cough tests among patients with neurodegenerative disorders. The proportion of patients with a normal cough reflex after the 1% citric acid challenge required was 28.6%. Assuming that the proportion of patients with a normal cough reflex after 8 weeks of therapy will be 50%, a sample size of 27 participants was estimated, based on an alpha level of 0.10, power of 0.80, and 10% dropout rate.

### 2.4 Data acquisition

Clinical evaluation and diagnosis will be confirmed by two neurologists (MN and HY). The following data will be recorded: body height, weight, body mass index, grip power, calf circumference, disease duration, past medical history, habits of alcohol drinking and smoking, blood pressure, heart rate, oxygen saturation, Unified Parkinson's Disease Rating Scale score [18], medication, Functional Oral Intake Scale score, Eating Assessment Tool-10 score, and blood test values [19]. Cough test, tongue pressure, and peak expiratory flow will be investigated as previously reported [5,20]. Moreover, novel methods, including multichannel surface electromyography, electronic stethoscope, and artificial intelligence system, will be applied. The data will be stored in the Redcap, Electronic Data Capture system. Author YH, who will be blinded to the acquired data, will independently monitor and audit the stored data every week.

### 2.5 Data analysis and statistical methods

Safety analysis will include an evaluation of the proportion of patients with skin symptoms and of exacerbation of neurological symptoms in the head and neck due to the electrical stimulation. If these symptoms are found, the therapy will be discontinued. To assess primary and secondary endpoints, the swallowing status of each patient at the time of the initial intervention will be compared with that at the end of the intervention (8 weeks from the initiation of the intervention) or at 8 weeks after the end of the intervention (16 weeks from intervention initiation). Statistical analysis will be performed using JMP statistical software version 16 (SAS Institute Inc., Cary, NC, USA). As appropriate, the statistical significance of intergroup differences will be assessed using χ^2^ test, Mann–Whitney *U* test, or unpaired *t*-test. We will consider p < 0.05 reflective of statistical significance.

### 2.6 Registration

This study will be performed from June 2022 to May 2024 at Hiroshima University Hospital. Trial Registration number: jRCTs062220013; pre-results.

## 3 Discussion

In this clinical trial, we will examine the usefulness of percutaneous neck interferential current sensory stimulation for patients with PD. Hypoesthesia of the pharynx and larynx is a particular characteristic of PD [4]; therefore, we will mainly measure the cough reflex as the primary endpoint. However, PD leads to various swallowing disorders, requiring the use of numerous instruments for evaluation. We will measure the tongue pressure to assess the oral cavity function because low tongue pressure indicates dysfunction of the oral phase of swallowing in many neurological diseases [20,21]. In patients with acute stroke, basal ganglia lesions are associated with delayed swallowing reflex [22]. The presence of abnormal basal ganglia is a characteristic of PD; thus, we will examine the delayed swallowing reflex via videofluoroscopic examination. Since interferential current stimulation could affect the central pattern generator and sensory nerve, an improvement in the swallowing reflex can be expected. Moreover, drooling or sialorrhea and dementia have attracted attention as clinical predictors of dysphagia in patients with PD [23]. Cervical interferential current stimulation is also effective for patients with dysphagia and dementia. We will determine the relevant risk factors for dysphagia in patients with PD and contributory factors of interferential current stimulation [24].

Moreover, this study will use novel instruments, such as multichannel surface electromyography and electronic stethoscope, to evaluate swallowing function in patients with PD. Multichannel surface electromyography can evaluate the recruitment of motor units and the effect of electrical stimulation [25,26]. Recently, electronic stethoscopes have been developed for the evaluation of swallowing sounds with the help of artificial intelligence [27]. These novel methods of evaluation can provide new insights on dysphagia in patients with PD and the usefulness of percutaneous neck interferential current stimulation. However, this exploratory study is limited by the single-arm, open-label design and small sample size.

## Funding

MN reports grants from 10.13039/501100001691Grants-in-Aid for Scientific Research (21K17512), the Tsuchiya Memorial Medical Foundation, Japanese Society of Dysphagia Rehabilitation, 10.13039/100008734Mitsui Sumitomo Insurance Welfare Foundation, and 10.13039/501100005936Casio Science Promotion Foundation.

YS reports grants from 10.13039/501100001691Grants-in-Aid for Scientific Research (21K10072).

HM reports grants from the 10.13039/501100001691Grants-in-Aid for Scientific Research (20K07887).

## Data statement

The data supporting this study's findings will be available from the corresponding author upon reasonable request.

## Declaration of competing interest

The authors declare that they have no known competing financial interests or personal relationships that could have appeared to influence the work reported in this paper.
